# Medication Adherence and Associated Factors in Patients With Type 2 Diabetes: A Structural Equation Model

**DOI:** 10.3389/fpubh.2021.730845

**Published:** 2021-11-04

**Authors:** Jing Huang, Shenglan Ding, Shuyuan Xiong, Zhiping Liu

**Affiliations:** ^1^Department of Endocrinology, The First Affiliated Hospital of Chongqing Medical University, Chongqing, China; ^2^Department of Nursing, Chengdu Women's and Children's Central Hospital, School of Medicine, University of Electronic Science and Technology of China, Chengdu, China

**Keywords:** neuroticism, social support, self-efficacy, medication adherence, type 2 diabetes, structural equation model

## Abstract

**Background:** The number of patients with type 2 diabetes (T2D) is increasing. Medication treatment is of great importance to stabilize blood glucose. Previous studies have reported that neuroticism, self-efficacy, and social support are factors associated with medication adherence, but few studies have fully investigated the mechanisms between these factors and medication adherence in patients with T2D.

**Purpose:** To explore the prevalence of medication adherence and the factors associated with medication adherence in patients with T2D.

**Methods:** A cross-sectional study consisting of 483 patients with T2D was conducted from July to December 2020. Questionnaires containing sociodemographic and clinical characteristics, the Morisky Medication Adherence Scale-8 (MMAS-8), the neuroticism subscale of the Eysenck Personality Questionnaire-Revised Short Scale (EPQ-RS), the Multidimensional Scale of Perceived Social Support (MSPSS), and the Diabetes Management Self-efficacy Scale (DMSES) were used to collect data. The structural equation model (SEM) was used to test the hypotheses.

**Results:** This study included 305 (63.1%) medication adherence and 178 (36.9%) medication non-adherence patients with T2D. Social support directly influenced medication adherence (β = 0.115, *P* = 0.029) and indirectly influenced medication adherence through self-efficacy (β = 0.044, *P* = 0.016). Self-efficacy directly influenced medication adherence (β = 0.139, *P* = 0.023). Neuroticism indirectly affected medication adherence through social support (β = −0.027, *P* = 0.023) and self-efficacy (β = −0.019, *P* = 0.014). Moreover, there was a sequential mediating effect of social support and self-efficacy on the relationship between neuroticism and medication adherence (β = −0.010, *P* = 0.012). After controlling for age and gender, similar results were obtained. The model fit indices showed a good fit.

**Conclusions:** The medication adherence of patients with T2D needs to be improved. Neuroticism, social support, and self-efficacy had direct or indirect effects on medication adherence in patients with T2D. Healthcare providers should comprehensively develop intervention programs based on neuroticism, social support, and self-efficacy to improve medication adherence in patients with T2D.

## Introduction

During the past three decades, the number of people with diabetes mellitus has continued to increase globally (1), and patients with type 2 diabetes (T2D) account for more than 90% of all patients with diabetes (2). As a complex and chronic disease, T2D not only brings serious physical and psychological distress to both patients and caregivers (2) but also causes a large economic burden to society (3). Therefore, the prevention and treatment of T2D is particularly important. Guidelines recommend that most patients with T2D should receive appropriate medical care when lifestyle changes can no longer achieve metabolic control (4, 5). Current evidence shows that intensive antihyperglycemic therapy can effectively reduce the incidence of diabetes complications and death (6). Therefore, medication adherence is important for achieving the treatment effect (7).

Medication adherence refers to taking medication according to medical advice and is assessed as the number of drugs taken or injected by a patient within a certain period of time being at least 80% of the number of drugs prescribed by the physician at the same time (8, 9). Better metabolic control (7), higher quality of life (10), and lower hospitalization rates (11) are associated with high medication adherence. However, inadequate medication adherence is very common among patients with T2D. Zhang et al. (12) found that 59.8% of patients with T2D had medication non-adherence. Raum et al. (13) found that the non-adherence rates of male and female patients with T2D were 24.0 and 23.0%, respectively; a poor glycemic control rate was found to be higher in patients reporting non-adherence than in patients reporting adherence. In another study (14), 21.2% of patients with T2D were assessed as having poor adherence. Medication adherence is an important factor influencing metabolic control, but it is not satisfactory. Thus, exploring factors associated with medication adherence in patients with T2D is essential to contribute to developing intervention programs to improve medication adherence and promote the health outcomes of patients with T2D.

Some psychological factors have been confirmed to be linked with medication adherence. First, the personality trait of neuroticism is a relatively stable tendency to make negative emotional responses to negative events (15). The behavior of people who are greatly affected by neuroticism tends to endanger their health (16), and these patients also report more side effects without corresponding physiological changes (17). Previous studies showed that neuroticism was negatively correlated with medication adherence in patients with chronic diseases (16), such as T2D (18). Therefore, we propose in hypothesis 1 that neuroticism directly affects medication adherence in patients with T2D. Second, social support generally refers to the care and encouragement that patients receive from their friends and families during the course of the disease (19). High levels of social support are associated with better health behaviors (20). The impact of changes in the level of social support on changes in the degree of medication adherence may be complex (21). Previous studies showed that social support was positively correlated with medication adherence in patients with T2D (22). Thus, we propose in hypothesis 2 that social support directly affects medication adherence in patients with T2D. Third, self-efficacy refers to the self-confidence of individuals using their own abilities to achieve a certain goal, and it affects the individual's response to the tasks and the way of thinking (23). Self-efficacy is related to health-related intentions and behavior (24). Previous studies showed a significant positive correlation between self-efficacy and medication adherence in patients with hypertension (23), HIV (25), and T2D (26). Therefore, we propose in hypothesis 3 that self-efficacy has a direct effect on medication adherence in patients with T2D.

Moreover, the above factors are interrelated. First, higher neuroticism was associated with lower self-efficacy (27). People with a higher degree of neuroticism are more sensitive to negative information and are more prone to self-doubt, which may lead to lower self-efficacy (28). Combined with the direct effects of neuroticism and self-efficacy on medication adherence, we propose hypothesis 4 that self-efficacy may play a mediating role in the relationship between neuroticism and medication adherence in patients with T2D. This is consistent with the hypothesis of Axelsson et al. (29), but their results showed that the relationship between self-efficacy and medication adherence was not significant in people with chronic disease. Second, neuroticism was identified as a significant predictor of social support (30). Personality factors may affect people's perception and response to supportive behaviors (31). Combined with the direct effects of neuroticism and social support on medication adherence, we propose in hypothesis 5 that social support may mediate the relationship between neuroticism and medication adherence in patients with T2D. Third, previous studies demonstrated that social support influenced self-efficacy directly (32). The establishment of supportive social relationships may promote self-belief and enhance self-efficacy (33). Moreover, another previous study showed that social support had an indirect effect on antiretroviral therapy adherence through self-efficacy in patients with HIV (25). Based on these accumulated findings, we propose in hypothesis 6 that self-efficacy may mediate the effect of social support on medication adherence in patients with T2D and in hypothesis 7 that neuroticism may indirectly affect medication adherence through the sequential mediating effects of social support and self-efficacy.

The current evidence suggests that patients with T2D may have the above hypothetical relationship; however, research focusing on this aspect is limited. Exploring the relationship between these factors and medication adherence is of vital importance to develop effective intervention programs to improve medication adherence in patients with T2D. Therefore, in this study, we aimed to test the above hypotheses to disentangle the various relationships between medication adherence, neuroticism, social support, and self-efficacy in patients with T2D.

## Methods

### Study Design and Participants

This was a cross-sectional study. From July to December 2020, the convenience sampling method was adopted to recruit patients from the inpatient departments of the First Affiliated Hospital of Chongqing Medical University in Chongqing, China. The inclusion criteria were as follows: (1) diagnosed with T2D for at least 1 year, (2) age ≥ 18 years, (3) currently receiving glucose-lowering therapy, and (4) conscious and voluntary. Patients whose condition was too severe to complete the survey (such as dyspnea, dizziness, and palpitation, etc.,) or patients with a history of mental illness were excluded from the study.

### Data Collection

The researchers received uniform training before the survey. Structured questionnaires were used to collect data. First, the researchers explained the study protocol to the patients. Second, the patients who were willing to take part in the survey signed a written informed consent. Third, the patients filled out the questionnaires independently after the researchers conducted unified guidance. When the patients encountered difficulties, the researchers wound provide assistance, such as detailed explanations and reading items. Finally, the researchers collected the questionnaires on the spot after the patients filled out them.

### Ethics Approval and Informed Consent

The study was approved by the ethics committee of the First Affiliated Hospital of Chongqing Medical University (2020–418), and written informed consent was obtained for each patient before participating in the study.

### Assessments

The questionnaire contained sociodemographic and clinical characteristics, such as age, gender, educational level, marital status, employment status, cohabitation status, duration of diabetes, and glucose-lowering therapy, as well as associated factors measured by instruments, such as medication adherence, neuroticism, social support, and self-efficacy. Among them, variables from patient's self-reports were educational level, marital status, employment status, cohabitation status, medication adherence, neuroticism, social support, and self-efficacy; variables from medical records included age, gender, duration of diabetes, and glucose-lowering therapy.

Medication adherence was assessed by the Morisky Medication Adherence Scale-8 (MMAS-8), which was developed by Morishy et al. (34–36). In this study, patients with MMAS-8 score ≥6 were classified as medication adherence. The MMAS-8 has been validated in the Chinese population (37). The Cronbach's α was 0.608 in this study.

Neuroticism was assessed by the neuroticism subscale of the Eysenck Personality Questionnaire-Revised Short Scale (EPQ-RS), which was developed by Eysenck et al. to evaluate the 4 domains of personality: neuroticism, extraversion, psychoticism, and a lie detector inventory (38). The scale is a 48-item questionnaire (“yes” = 1 point, “no” = 0 points). The total score of the neuroticism subscale ranges from 0 to 12. The higher the score, the more obvious the neuroticism characteristics. The EPQ-RS has been validated in the Chinese population (39). The Cronbach's α of the neuroticism subscale in our sample was 0.838.

Social support was assessed by the Multidimensional Scale of Perceived Social Support (MSPSS), which was developed by Zimet et al. (40). It consists of 12 items and evaluates perceived social support from 3 different domains, including family support, friend support, and other support, with 4 items in each dimension. The scale uses a 7-point Likert scoring method (“very strongly disagree” = 1 point, “very strongly agree” = 7 points), with a total score ranging from 12 to 84. The higher the score, the better the level of social support that an individual feels subjectively. The MSPSS has been validated in the Chinese population (41). The Cronbach's α in our study was 0.926.

Self-efficacy was assessed by the Diabetes Management Self-efficacy Scale (DMSES), which was developed by Bijl et al. (42). The scale contains 20 items divided into 4 domains: nutrition, physical exercise and weight, blood glucose and feet check, and medical treatment. The total score of the DMSES ranges from 0 to 200 (“totally impossible” = 0 points, “completely possible” = 10 points). The higher the score, the greater the self-efficacy. The DMSES has been validated in the Chinese population (43). The Cronbach's α was 0.934 in this study.

### Statistical Analysis

**Figure 1:**
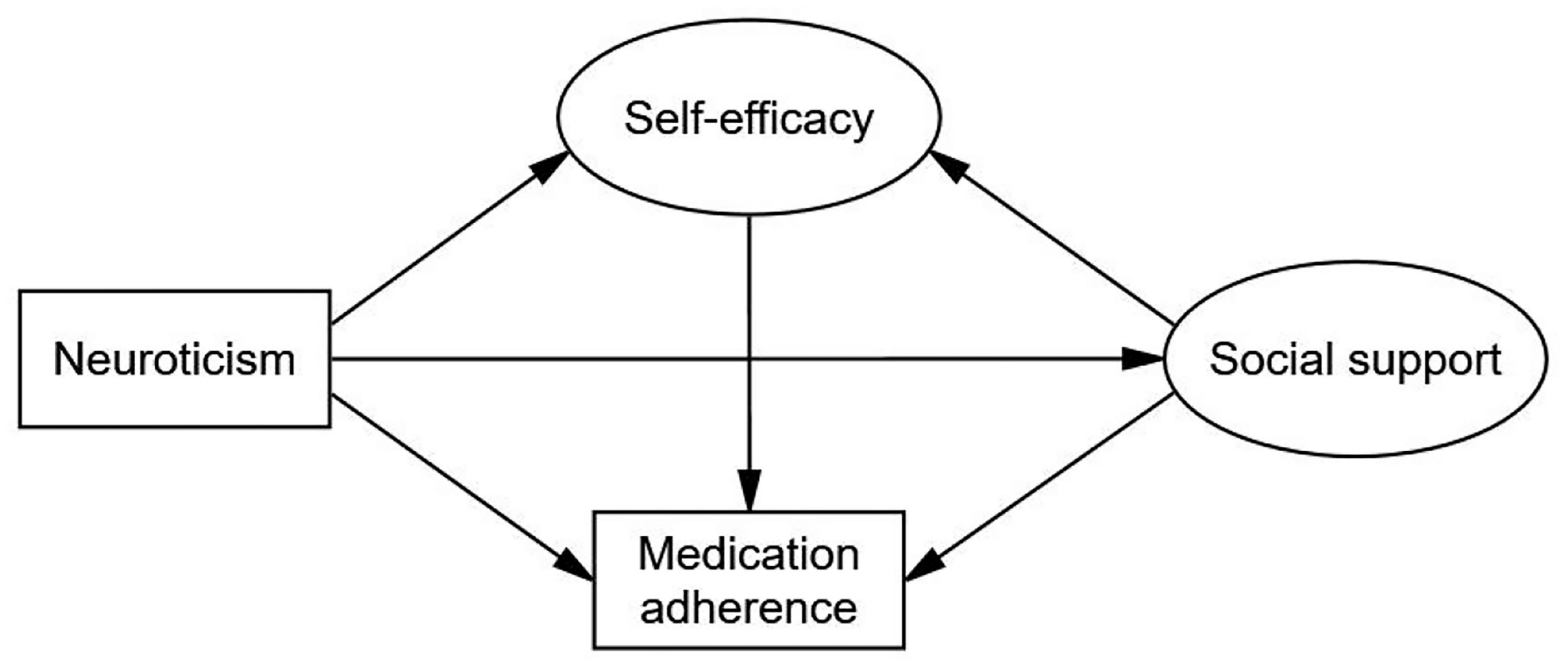
Conceptual model. Circles indicate latent variables. Rectangles indicate exogenous variables. The MMAS-8 Scale, content, name, and trademarks are protected by US copyright and trademark laws. Permission for use of the scale and its coding is required. A license agreement is available from MMAR, LLC., Donald E. Morisky, ScD, ScM, MSPH, 294 Lindura Ct., USA; donald.morisky@moriskyscale.com.

Data analyses were conducted in IBM SPSS 23.0 (IBM Corporation, Armonk, State of New York, USA) and IBM SPSS Amos 23.0 (IBM Corporation, Armonk, State of New York, USA). For continuous data, variables were described as the means and standard deviations (SDs) or medians and interquartile ranges (IQRs), and the differences between groups were compared using independent sample *t*-tests or Mann-Whitney U tests. For categorical data, variables were described as counts and percentages, and the differences between groups were compared using χ^2^ tests or Fisher's exact tests. Spearman correlations were used to examine the associations between neuroticism, social support, self-efficacy, and medication adherence. A structural equation model (SEM) with maximum likelihood estimation was used to test the hypotheses outlined in the conceptual model (Figure 1). The non-parametric bootstrapping method can be used to make the model estimate more stable and test the significance of indirect effects (44). Thus, we used the bootstrapping method with 5,000 samples for testing (44). This method computed bias-corrected 95% confidence interval (CI) and percentile 95% CI, and a result was considered significant if the 95% CI excluded zero. Individuals with missing data were deleted. The incremental fit index (IFI), comparative fit index (CFI), and normed fit index (NFI) ≥0.90, and root mean squared error of approximation (RMSEA) ≤ 0.08 were used to confirm the model fit (45, 46).

## Results

### Participant Characteristics

A total of 494 patients participated in the survey, of which 11 had missing data, and 483 patients were included in the analysis. The sociodemographic and clinical characteristics of the patients are presented in Table 1. The majority of them were male (290, 60.0%), had a high school or below educational level (312, 64.6%), were married (420, 87.0%), were not employed (330, 68.3%), lived with others (443, 91.7%), had had diabetes for 5–14 years (214, 44.3%), and used glucose-lowering agents alone (243, 50.3%). The proportion of females among adherent patients was higher than that among non-adherent patients (*P* = 0.020). The average age of the patients was 60.22 ± 11.51 years old, and the age of adherent patients was greater than that of non-adherent patients (*P* = 0.028). The proportion of patients who were not employed among adherent patients was higher than that among non-adherent patients. High social support (*P* = 0.007), high self-efficacy (*P* = 0.025) and low neuroticism (*P* = 0.001) were associated with medication adherence.

**Table 1:** Sociodemographic and clinical characteristics.

**Characteristics**	**Total, 483**	**Adherence, 305 (63.1)**	**Non-adherence, 178 (36.9)**	***P*** **-value**
Age, mean ± SD	60.22 ± 11.51	61.10 ± 11.59	58.71 ± 11.24	0.028**
Gender, *N* (%)				0.020*
Male	290 (60.0)	171 (56.1)	119 (66.9)	
Female	193 (40.0)	134 (43.9)	59 (33.1)	
Educational level, *N* (%)				0.238
High school or below	312 (64.6)	203 (66.6)	109 (61.2)	
Undergraduate or above	171 (35.4)	102 (33.4)	69 (38.8)	
Marital status, *N* (%)				0.289
Married	420 (87.0)	269 (88.2)	151 (84.8)	
Single, divorced or other	63 (13.0)	36 (11.8)	27 (15.2)	
Employment status, *N* (%)				0.011*
Employed	153 (31.7)	84 (27.5)	69 (38.8)	
Other (unemployed, retired, etc.,)	330 (68.3)	221 (72.5)	109 (61.2)	
Cohabitation status, *N* (%)				0.929
Living with others	443 (91.7)	280 (91.8)	163 (91.6)	
Living alone	40 (8.3)	25 (8.2)	15 (8.4)	
Duration of diabetes, *N* (%)				0.659
<5	112 (23.2)	67 (22.0)	45 (25.3)	
5-14	214 (44.3)	139 (45.6)	75 (42.1)	
≥15	157 (32.5)	99 (32.5)	58 (32.6)	
Glucose-lowering therapy, *N* (%)				0.203
Agents alone^a^	243 (50.3)	151 (49.5)	92 (51.7)	
Insulin alone	51 (10.6)	38 (12.5)	13 (7.3)	
Insulin and agents	189 (39.1)	116 (38.0)	73 (41.0)	
Neuroticism, median (IQR)	2.0 (0.0–4.0)	1.0 (0.0–3.0)	2.0 (1.0–5.0)	0.001**
Social support, median (IQR)	65.0 (59.0–72.0)	66.0 (60.0–72.0)	64.0 (56.8–71.0)	0.007**
Self-efficacy, median (IQR)	160.0 (144.0–178.0)	163.0 (145.5–178.0)	156.5 (139.0–177.0)	0.025*
Medication adherence, median (IQR)	2.0 (1.0–2.0)	–	–	–

a *Glucose-lowering agents included both oral agents and injectable agents. SD, standard deviation; IQR, interquartile range; N, number*.

*Significant codes*:

** *P < 0.001*;

* *P < 0.05*.

### Bivariate Analyses

Correlations between medication adherence, neuroticism, social support, self-efficacy, age, and gender are shown in Table 2. Neuroticism was negatively associated with medication adherence (*r* = −0.168, *P* < 0.001), while social support (*r* = 0.167, *P* < 0.001) and self-efficacy (*r* = 0.157, *P* < 0.001) were positively related to medication adherence. Neuroticism was negatively correlated with social support (*r* = −0.218, *P* < 0.001) and self-efficacy (*r* = −0.192, *P* < 0.001). Social support was positively associated with self-efficacy (*r* = 0.340, *P* < 0.001).

**Table 2:** Spearman's correlation matrix of the study variables.

	**Age**	**Gender**	**Neuroticism**	**Social support**	**Self-efficacy**	**Medication adherence**
1. Age	1					
2. Gender	0.201***	1				
3. Neuroticism	−0.001	0.133**	1			
4. Social support	−0.062	−0.006	−0.218***	1		
5. Self-efficacy	0.004	−0.009	−0.192***	0.340***	1	
6. Medication adherence	0.051	0.061	−0.168***	0.167***	0.157**	1

*Significant codes*:

*** *P < 0.001*;

** *P < 0.01*.

### Structural Equation Model

**Figure 2:**
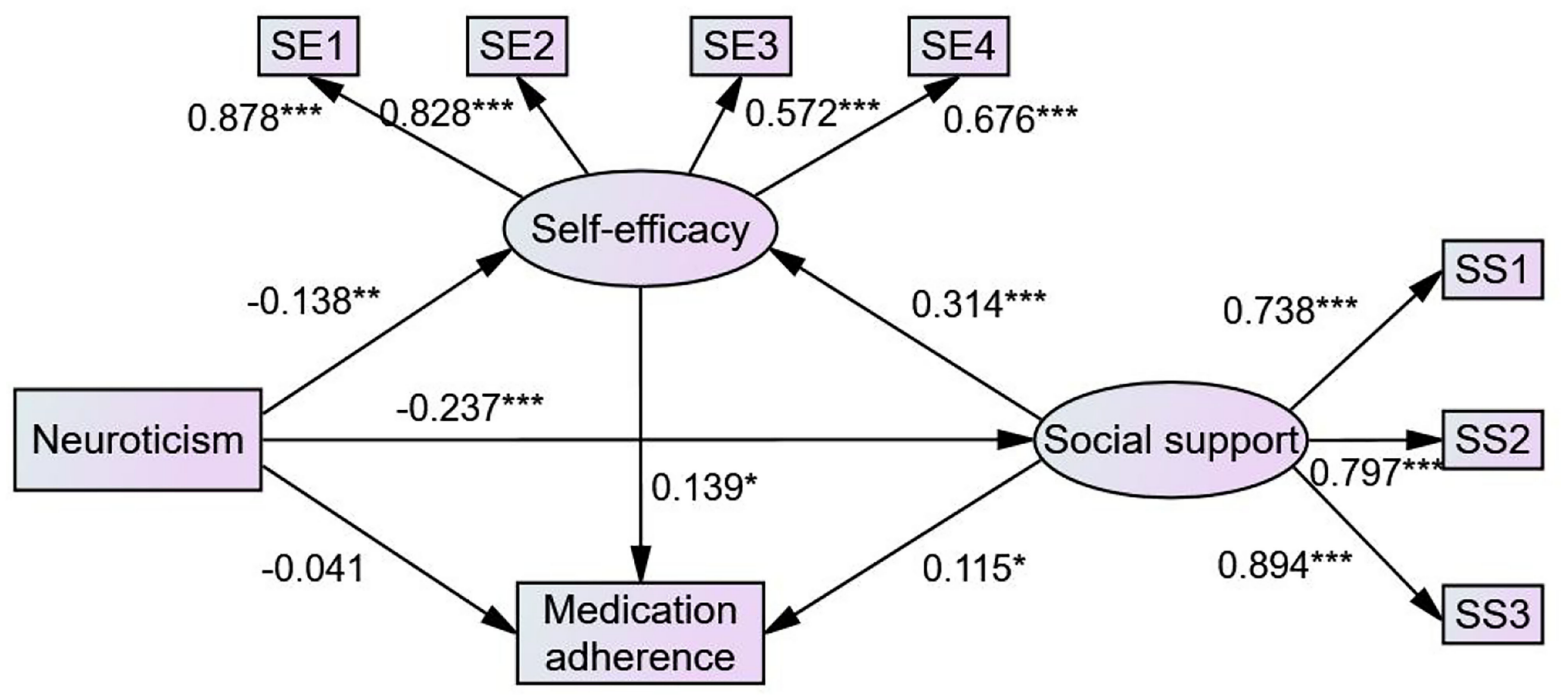
The final model of medication adherence in patients with type 2 diabetes (model 1). Circles indicate latent variables. Rectangles indicate exogenous variables. All path coefficients were standardized. Significant codes: ***P < 0.001; **P < 0.01; *P < 0.05.

**Figure 3:**
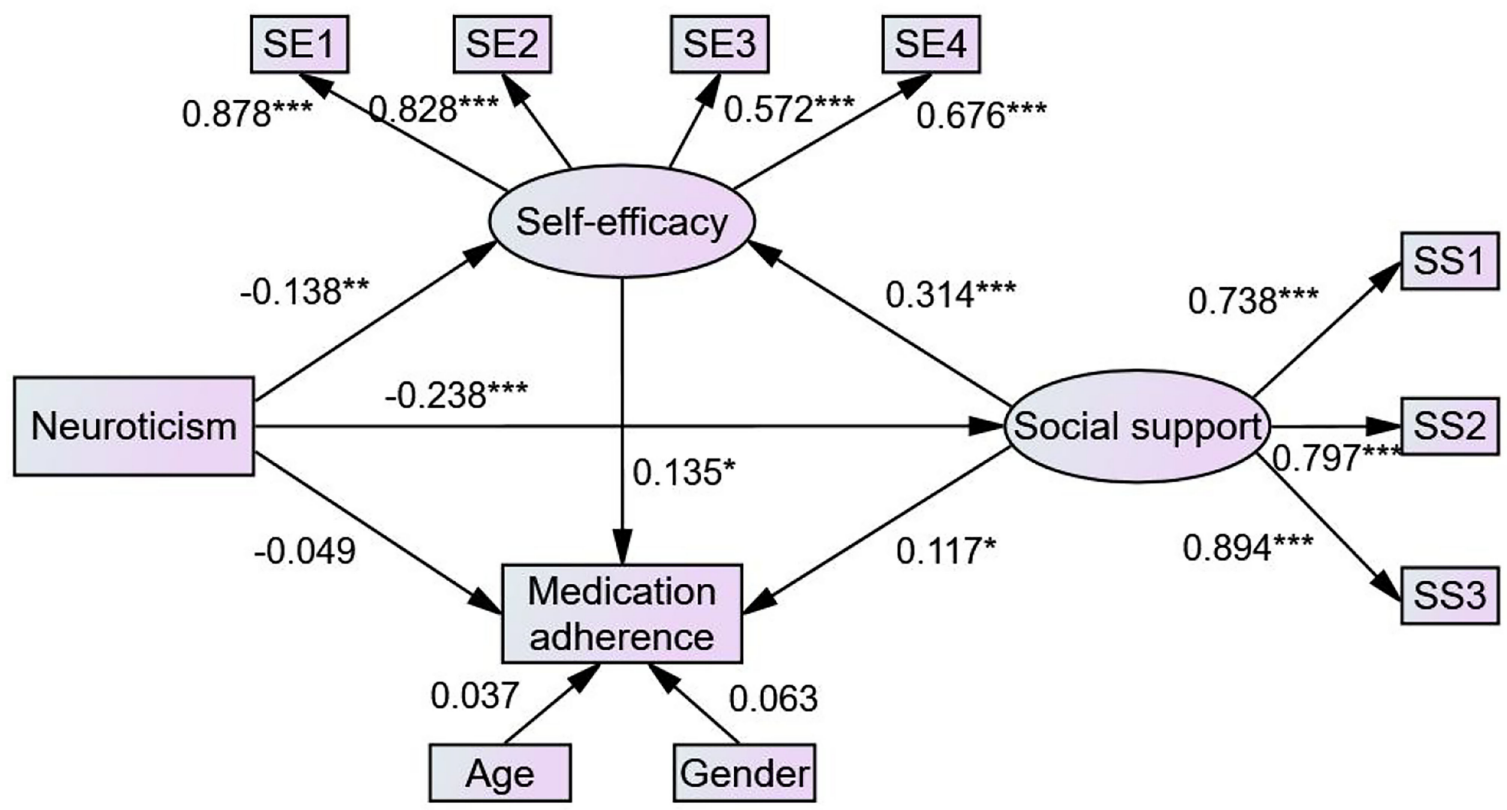
The final model of medication adherence in patients with type 2 diabetes (model 2). Circles indicate latent variables. Rectangles indicate exogenous variables. All path coefficients were standardized. Age and gender were controlled. Significant codes: ***P < 0.001; **P < 0.01; *P < 0.05.

The structural model in this study contained two observed variables and two latent variables. Figure 2 (model 1) illustrates the results of the SEM used to test the hypotheses of this study. Standardized direct, indirect, and total effects are shown in Table 3. Neuroticism had a direct effect on self-efficacy (β = −0.138, *P* = 0.006) and social support (β = −0.237, *P* < 0.001). Social support had a direct effect on self-efficacy (β = 0.314, *P* < 0.001) and medication adherence (β = 0.115, *P* = 0.029), supporting hypothesis 2. Self-efficacy had a direct effect on medication adherence (β = 0.139, *P* = 0.023), supporting hypothesis 3. While neuroticism did not directly influence medication adherence (β = 0.041, *P* = 0.383), it had indirect effects on medication adherence through social support (β = −0.027, *P* = 0.023) and self-efficacy (β = −0.019, *P* = 0.014), and social support and self-efficacy had a sequential mediating effect on the relationship between neuroticism and medication adherence (β = −0.010, *P* = 0.012), supporting hypotheses 4, 5, and 7. Social support also indirectly affected medication adherence *via* self-efficacy (β = 0.044, *P* = 0.016), supporting hypothesis 6. Furthermore, neuroticism indirectly influenced self-efficacy through social support (β = −0.075, *P* < 0.001). Therefore, all hypotheses were supported. It is worth noting that social support and self-efficacy played a full mediating role in the relationship between neuroticism and medication adherence. In model 1, the model fit was confirmed with the following four indicators (IFI = 0.960, CFI = 0.960, NFI = 0.946, and RMSEA = 0.075). After controlling for age and gender, we obtained similar results, as shown in Figure 3 (model 2) and Table 4. In model 2, the model fit was also confirmed with the following four indicators (IFI = 0.925, CFI = 0.924, NFI = 0.902, and RMSEA = 0.080).

**Table 3:** Direct, indirect, and total effects of the study variables in model 1.

	**β**	**SE**	**Percentile 95% CI**		**Bias-corrected** **percentile 95% CI**		***P*-value**
			**Lower**	**Upper**	**Lower**	**Upper**	
**Standardized direct effect**							
Neuroticism → Medication adherence	−0.041	0.047	−0.135	0.053	−0.133	0.054	0.383
Self-efficacy → Medication adherence	0.139	0.061	0.018	0.258	0.018	0.257	0.023*
Social support → Medication adherence	0.115	0.053	0.01	0.218	0.01	0.219	0.029*
Neuroticism → Self-efficacy	−0.138	0.049	−0.232	−0.039	−0.234	−0.041	0.006**
Social support → Self-efficacy	0.314	0.062	0.193	0.435	0.188	0.433	<0.001***
Neuroticism → Social support	−0.237	0.047	−0.327	−0.147	−0.328	−0.147	<0.001***
**Standardized indirect effect**							
Neuroticism → Self-efficacy → Medication adherence	−0.019	0.012	−0.046	−0.001	−0.051	−0.003	0.014*
Neuroticism → Social support → Medication adherence	−0.027	0.014	−0.058	−0.002	−0.06	−0.003	0.023*
Neuroticism → Social support → Self-efficacy → Medication adherence	−0.01	0.006	−0.023	−0.001	−0.026	−0.002	0.012*
Social support → Self-efficacy → Medication adherence	0.044	0.022	0.005	0.092	0.007	0.094	0.016*
Neuroticism → Social support → Self-efficacy	−0.075	0.022	−0.124	−0.037	−0.126	−0.039	<0.001***
**Standardized total effect**							
Neuroticism → Medication adherence	−0.098	0.044	−0.185	−0.012	−0.183	−0.009	0.030*
Social support → Medication adherence	0.158	0.052	0.057	0.26	0.056	0.26	0.003**
Neuroticism → Self-efficacy	−0.212	0.047	−0.302	−0.119	−0.302	−0.118	<0.001***

*Standardized estimating of 5,000 bootstrap sample. SE, standard error; CI, confidence interval*.

*Significant codes*:

*** *P < 0.001*;

** *P < 0.01*;

* *P < 0.05*.

**Table 4:** Direct, indirect, and total effects of the study variables in model 2.

	**β**	**SE**	**Percentile 95% CI**		**Bias-corrected percentile 95% CI**		***P*** **-value**
			**Lower**	**Upper**	**Lower**	**Upper**	
**Standardized direct effect**							
Neuroticism → Medication adherence	−0.049	0.048	−0.144	0.047	−0.145	0.046	0.297
Self-efficacy → Medication adherence	0.135	0.06	0.014	0.253	0.016	0.254	0.028*
Social support → Medication adherence	0.117	0.053	0.011	0.222	0.011	0.222	0.026*
Neuroticism → Self-efficacy	−0.138	0.049	−0.232	−0.039	−0.234	−0.041	0.006**
Social support → Self-efficacy	0.314	0.062	0.193	0.435	0.188	0.433	<0.001***
Neuroticism → Social support	−0.238	0.047	−0.328	−0.147	−0.328	−0.147	<0.001***
**Standardized indirect effect**							
Neuroticism → Self-efficacy → Medication adherence	−0.019	0.012	−0.045	−0.001	−0.05	−0.003	0.018*
Neuroticism → Social support → Medication adherence	−0.028	0.015	−0.059	−0.002	−0.061	−0.003	0.021*
Neuroticism → Social support → Self-efficacy → Medication adherence	−0.01	0.006	−0.023	−0.001	−0.025	−0.002	0.016*
Social support → Self-efficacy → Medication adherence	0.043	0.022	0.004	0.089	0.006	0.092	0.021*
Neuroticism → Social support → Self-efficacy	−0.075	0.022	−0.124	−0.037	−0.126	−0.039	<0.001***
**Standardized total effect**							
Neuroticism → Medication adherence	−0.106	0.045	−0.194	−0.018	−0.193	−0.017	0.021*
Social support → Medication adherence	0.159	0.052	0.057	0.262	0.055	0.26	0.003**
Neuroticism → Self-efficacy	−0.212	0.047	−0.302	−0.119	−0.302	−0.118	<0.001***

*Standardized estimating of 5,000 bootstrap sample. Age and gender were controlled. SE, standard error; CI, confidence interval*.

*Significant codes*:

*** *P < 0.001*;

** *P < 0.01*;

* *P < 0.05*.

## Discussion

This study provided evidence for the relationship among neuroticism, social support, self-efficacy, and medication adherence in patients with T2D and provided a theoretical basis for understanding factors that may improve medication adherence in patients with T2D. The SEM demonstrated that social support and self-efficacy were important predictors of medication adherence in patients with T2D, while neuroticism by itself was not a predictor of medication adherence in patients with T2D. Specifically, neuroticism had a direct effect on social support and self-efficacy and had an indirect effect on medication adherence through social support and self-efficacy. More notably, there was a sequential mediating effect of social support and self-efficacy on the association between neuroticism and medication adherence. In addition, social support had a direct impact on self-efficacy and had an indirect impact on medication adherence through self-efficacy. In our study, the proportion of participants with a medication adherence score <6 was 36.9%, showing inadequate medication adherence in patients with T2D, which was consistent with previous studies (12, 14). Improving medication adherence in patients with T2D is a significant problem that urgently needs to be addressed.

Social support was an important predictor of medication adherence in patients with T2D. Our results were consistent with previous studies showing that social support was positively correlated with medication adherence in patients with heart failure, psychosis, and HIV (21, 47, 48). A meta-analysis showed that functional social support referring to the help and encouragement that is provided to individuals by their social network played an important role in medication adherence (49). Higher social support is related to better psychological health and better health outcomes (50, 51), indicating that higher social support has more positive emotion and health behaviors, which may be an explanation for why higher social support is associated with better adherence to some extent. Furthermore, in a qualitative study, patients reported that their spouses or other caregivers provided practical support by directly helping and supervising their medication, which is important to increase their medication adherence (52). The practical support of a social network may be another explanation of the effect of social support on medication adherence. Therefore, enhancing social support from multiple sources, including society, family, and healthcare providers, is necessary to address insufficient medication adherence in patients with T2D.

In addition, self-efficacy was also an important predictor of medication adherence in patients with T2D, which was in line with previous studies showing that self-efficacy was positively correlated with medication adherence in patients with hypertension, HIV and diabetes (23, 25, 26). Patients with high self-efficacy may be more confident of good health outcomes resulting from medication adherence and thus more likely to adhere to medication (53). Moreover, self-efficacy was also a mediating variable in the influence of neuroticism and social support on medication adherence in patients with T2D in the final model. Possible explanations for the indirect effects may be as follows: (1) patients with high neuroticism are more likely to capture negative information and develop psychological problems, leading to low self-efficacy, which further leads to poor adherence; and (2) patients with high social support have high self-efficacy through their perception of social support and the encouragement and support of social networks and thus have more ability and confidence to persist in medication use. In previous studies, self-efficacy was also a mediator in the relationship between some health-related factors and health behaviors, such as medication literacy and medication adherence, diabetes distress and medication adherence, and HIV disclosure and medication adherence (23, 25, 26). The direct effects and moderating roles of self-efficacy further prove that self-efficacy plays a vital role in medication adherence in patients with T2D. Therefore, self-efficacy was a meaningful target for improving medication adherence in patients with T2D.

In our conceptual model, neuroticism was an important predictor of medication adherence in patients with T2D, and previous studies supported this hypothesis (18, 54). Our final model did not find a significant direct effect of neuroticism on medication adherence, indicating that social support and self-efficacy were full mediators in the association between neuroticism and medication adherence. A previous study found that there was no relationship between adherence to antidepressants and personality traits assessed through electronic drug use monitoring. Different results may be due to differences in samples and evaluation tools, which explains the complexity of the relationship between neuroticism and medication adherence to some extent (55). Specifically, in this study, the indirect effect of neuroticism on medication adherence included three main aspects. First, as mentioned earlier, patients with high neuroticism scores may have less confidence in the treatment, leading to insufficient adherence. Second, neurotic people are less likely to perceive others in their social networks as sources of social support (31), so patients with high neuroticism scores perceive low social support, leading to poor adherence. Third, combined with the mediating effects of self-efficacy in the relationship between social support and medication adherence, the sequential mediating effects of social support and self-efficacy on the relationship between neuroticism and medication adherence may be explained as follows: neurotic personality affects patient's recognition and perception of social support, and actual and perceived social support affects the patient's ability and confidence to adhere to medication, which ultimately affects medication adherence. Therefore, the potential explanation for why neuroticism is negatively associated with medication adherence may be the mediating role of social support and self-efficacy.

This study clarified the relationship among medication adherence, neuroticism, social support, and self-efficacy and provided meaningful information for healthcare providers of patients with T2D. However, this study has some limitations. First, the cross-sectional design of this survey limited our ability to develop causal statements about the relationships between study variables. In the future, a longitudinal design should be used to verify causality. Second, self-report questionnaires were used to collect data in this study, which inevitably led to reporting and recall bias. Thus, more objective adherence monitoring methods should be adopted in the future. Third, because our convenience sample was only from China, we should be careful to extend our conclusions to other populations. Therefore, researchers are encouraged to verify the conclusions in different populations to ensure the reasonableness and stability of the theory.

To our knowledge, this is the first study to explore the effects of neuroticism, social support, and self-efficacy on medication adherence in patients with T2D *via* SEM. Our results provide practical guidance for healthcare providers of patients with T2D. This model suggests that the development of intervention programs to improve medication adherence in patients with T2D should be considered from multiple perspectives. First, social support is a key factor for medication adherence in patients with T2D. It not only directly affects adherence but also indirectly affects adherence through self-efficacy. Therefore, measures aiming at enhancing social support for patients with T2D should be implemented in intervention programs, thereby improving medication adherence in patients with T2D. Second, to improve medication adherence in patients with T2D, in addition to ensuring the beneficial conditions of the external environment, we should also focus on their inherent ability and self-confidence. Therefore, healthcare providers should pay more attention to self-efficacy and take measures to strengthen the self-efficacy of patients with T2D to improve their medication adherence. Third, for patients with obvious neurotic personality tendencies, more attention should be given to the improvement of social support and self-efficacy, thereby reducing the negative effects of neuroticism on medication adherence. Finally, the development of intervention measures should be individualized. In addition, the proportion of non-adherent individuals was higher among patients who were relatively young, male, and employed. Thus, healthcare providers should pay more attention to these groups to greatly improve the medication adherence in patients with T2D.

## Conclusions

This study identified some factors associated with medication adherence in patients with T2D, clarified the relationship between these factors, provided a new perspective for intervention studies associated with medication adherence, and provided a theoretical basis for the development of intervention programs aiming to improve medication adherence in patients with T2D. Neuroticism, social support, and self-efficacy directly or indirectly influenced medication adherence in patients with T2D; therefore, healthcare providers should focus on these aspects when developing intervention programs.

## Data Availability Statement

The raw data supporting the conclusions of this article will be made available by the authors, without undue reservation.

## Ethics Statement

The studies involving human participants were reviewed and approved by the Ethics Committee of the First Affiliated Hospital of Chongqing Medical University (No. 2020–418). The patients/participants provided their written informed consent to participate in this study.

## Author Contributions

ZL was responsible for checking the manuscript and proposed the research direction. JH, SD, and SX were responsible for questionnaire development and data collection. JH was responsible for statistical analysis. JH and SD were responsible for writing the manuscript. All authors contributed to the article and approved the submitted version.

## Funding

This work was supported by the Nursing Research Fund of Chongqing Medical University (2019hlxk07).

## Conflict of Interest

The authors declare that the research was conducted in the absence of any commercial or financial relationships that could be construed as a potential conflict of interest.

## Publisher's Note

All claims expressed in this article are solely those of the authors and do not necessarily represent those of their affiliated organizations, or those of the publisher, the editors and the reviewers. Any product that may be evaluated in this article, or claim that may be made by its manufacturer, is not guaranteed or endorsed by the publisher.

## Abbreviations

T2D: type 2 diabetes
MMAS-8: the Morisky Medication Adherence Scale-8
EPQ-RS: the Eysenck Personality Questionnaire-Revised Short Scale
MSPSS: the Multidimensional Scale of Perceived Social Support
DMSES: the Diabetes Management Self-efficacy Scale
SEM: structural equation model
SDs: standard deviations
IQRs: interquartile ranges
CI: confidence interval
IFI: incremental fit index
CFI: comparative fit index
NFI: normed fit index
RMSEA: root mean squared error of approximation
N: number
SE: standard error.
